# Heterogeneity of autoantibodies in 100 patients with autoimmune myositis: insights into clinical features and outcomes

**DOI:** 10.1186/ar2276

**Published:** 2007-08-09

**Authors:** Martial Koenig, Marvin J Fritzler, Ira N Targoff, Yves Troyanov, Jean-Luc Senécal

**Affiliations:** 1 University of Montreal School of Medicine, and Laboratory for Research in Autoimmunity, Centre Hospitalier de l'Université de Montréal, M-4243, 1560 East Sherbrooke Street, Montreal, Quebec, Canada H2L 4M1; 2 Faculty of Medicine HRB409, University of Calgary, 3330 Hospital Dr. NW, Calgary, Alberta, Canada T2N 4N1; 3 Arthritis and Immunology, University of Oklahoma Health Sciences Center, 825 NE 13th Street Oklahoma City, OK 73104, and Oklahoma Medical Research Foundation, Oklahoma City, Oklahoma, USA

## Abstract

The objective of this study was to determine the prevalence, mutual associations, clinical manifestations, and diagnoses associated with serum autoantibodies, as detected using recently available immunoassays, in patients with autoimmune myositis (AIM). Sera and clinical data were collected from 100 patients with AIM followed longitudinally. Sera were screened cross-sectionally for 21 autoantibodies by multiplex addressable laser bead immunoassay, line blot immunoassay, immunoprecipitation of *in vitro *translated recombinant protein, protein A assisted immunoprecipitation, and enzyme-linked immunosorbent assay. Diagnoses were determined using the Bohan and Peter classification as well as recently proposed classifications. Relationships between autoantibodies and clinical manifestations were analyzed by multiple logistic regression. One or more autoantibodies encompassing 19 specificities were present in 80% of the patients. The most common autoantibodies were anti-Ro52 (30% of patients), anti-Ku (23%), anti-synthetases (22%), anti-U1RNP (15%), and anti-fibrillarin (14%). In the presence of autoantibodies to Ku, synthetases, U1RNP, fibrillarin, PM-Scl, or scleroderma autoantigens, at least one more autoantibody was detected in the majority of sera and at least two more autoantibodies in over one-third of sera. The largest number of concurrent autoantibodies was six autoantibodies. Overall, 44 distinct combinations of autoantibodies were counted. Most autoantibodies were unrestricted to any AIM diagnostic category. Distinct clinical syndromes and therapeutic responses were associated with anti-Jo-1, anti-fibrillarin, anti-U1RNP, anti-Ro, anti-Ro52, and autoantibodies to scleroderma autoantigens. We conclude that a significant proportion of AIM patients are characterized by complex associations of autoantibodies. Certain myositis autoantibodies are markers for distinct overlap syndromes and predict therapeutic outcomes. The ultimate clinical features, disease course, and response to therapy in a given AIM patient may be linked to the particular set of associated autoantibodies. These results provide a rationale for patient profiling and its application to therapeutics, because it cannot be assumed that the B-cell response is the same even in the majority of patients in a given diagnostic category.

## Introduction

Autoimmune myositis (AIM) is a syndrome characterized by involvement of the cellular and humoral immune systems in skeletal muscle pathology, immunogenetic modulation, response to immunotherapies, and the presence of autoantibodies in the serum of many patients [1,2]. Although AIM is commonly classified using the original 1975 classification proposed by Bohan and Peter [3,4], this approach has become subject to increasing debate [5-7]. The Bohan and Peter classification has been criticized for over-diagnosing polymyositis (PM) [8]; for loosely defining myositis in overlap (overlap myositis [OM]) with another connective tissue disease (CTD) [9]; for clinical, genetic, and immunologic heterogeneity in all subsets [10]; and for being obsolete [11]. The discovery of myositis specific antibodies (MSAs) and myositis-associated antibodies (MAAs) led to a serologic approach complementary to the Bohan and Peter classification, because striking associations of MSAs with clinical features, immunogenetics, and survival were observed [10].

However, this approach has been limited by several constraints. First, until recently, sophisticated methods that are costly, labor intensive, and not always routinely available were required for identification of most MSAs, limiting their widespread use. Second, because MSAs are relatively insensitive markers for myositis [12], this serologic approach led to the creation of a large and heterogeneous group of MSA-negative patients, who were undefined with respect to diagnosis, prognosis, and survival [13]. Third, the emphasis on MSAs has resulted in a common perception among clinicians that AIM is characterized by the presence of single autoantibody specificities, whereas associations between an MSA and MAAs are not uncommon. However, the interrelationships between these sets of autoantibodies and their clinical impact have yet not been explored in depth. Taken together, these constraints identify a need to develop more sensitive and less costly methods for detecting MSAs and MAAs, and for analyzing the interrelationships between these autoantibodies.

As a step toward resolving these issues, and with the objective of improving AIM classifications, in this study we focus on AIM autoantibodies by conducting an in-depth examination of their prevalence, distribution and mutual associations, as well as their corresponding diagnoses and clinical manifestations. We took stock from our recently proposed novel approach to the classification of AIM, which brings together strong clinical evidence of myositis that is readily identifiable by clinicians and the diagnostic value of MSA and MAA tests [14]. In the present report, we examine the same cohort for an expanded panel of 21 autoantibodies to major AIM autoantigens, using recently available line immunoassay (LIA) and addressable laser bead multiplex technologies. We also used multiple logistic regression analysis to identify clinical manifestations independently associated with these autoantibodies.

## Patients and methods

### Patients and data collection

We conducted a cross-sectional serum study of 100 adult French Canadian patients with AIM, followed longitudinally, who were seen up to 2001 at the Centre Hospitalier de l'Université de Montréal, a tertiary care center. A list of AIM patients was obtained from the medical records using discharge summary diagnostic codes corresponding to PM, dermatomyositis (DM), myositis, mixed CTD, and overlap syndrome. The five inclusion criteria were as follows. First, only French Canadian patients were eligible. Second, the illness fulfilled Bohan and Peter criteria for definite, probable, or possible PM or DM by the end of follow up [3,4]. Patients with possible PM were included because this diagnosis is not uncommon in clinical practice and the prolonged follow up provided an opportunity to examine its outcome. Third, patients had to be 18 years or older at diagnosis of myositis (therefore juvenile DM, as defined by Bohan and Peter, was excluded). Fourth, inclusion body myositis, rare forms of AIM, and non-AIM causes of myopathy (such as muscular dystrophies) were excluded. Also excluded were patients diagnosed as having AIM in whom a non-AIM myopathy was ultimately diagnosed upon follow up. Finally, a frozen serum sample had to be available for immunologic studies. At myositis diagnosis, according to the diagnostic criteria proposed by Bohan and Peter [3,4], 36 definite, 45 probable, and 18 possible cases of myositis were seen, and a single patient had a DM rash and a myopathic electromyogram. At last follow up, there were 47 definite, 41 probable, and 12 possible cases of myositis, and detailed features of these patients have been described elsewhere [14].

Data on history, physical findings, and laboratory investigations were obtained by retrospective medical record review using a standardized protocol. Written consent was obtained from treating physicians to communicate with and examine the patients for further data collection. The project was approved by the Centre Hospitalier de l'Université de Montréal research and ethics committees. The diagnoses of myositis were made at Centre Hospitalier de l'Université de Montréal in 87 patients, and 13 additional patients were referred with an established AIM diagnosis. A muscle biopsy and an electromyogram were done in 87 and 88 patients, respectively. Among the 13 patients in whom no muscle biopsy was taken, 12 (92%) patients had a DM rash (*n *= 8) and/or overlap CTD features (*n *= 7). Five patients had possible myositis, three had probable myositis, and five had definite myositis. Definitions for overlap CTD features, target organ involvement, and clinical characteristics are described in detail elsewhere [14] and are summarized in Table 1. All living patients (*n *= 77) but one were examined or contacted by us between June 1999 and April 2001. The associations between autoantibodies and clinical features were determined at myositis diagnosis, whereas associations with myositis course and response to therapy were based on cumulative data at last follow up. Definitions for monophasic myositis, response to prednisone alone, and need for a second immunosuppressive drug were as described previously [14].

**Table 1:** Description of three classifications for autoimmune myositis

Classification	Abbreviation	Definition/description
Original Bohan and Peter classification	PM	Primary polymyositis
	DM	Primary dermatomyositis
	CTM	Myositis with another connective tissue disease
	CAM	Myositis associated with cancer
Modified Bohan and Peter classification	PM	Pure polymyositis
	DM	Pure dermatomyositis
	OM	Overlap myositis: with at least one clinical overlap feature^a^
	CAM	Cancer-associated myositis: with clinical paraneoplasic features^b^
Novel clinicoserologic classification	PM	Pure polymyositis
	DM	Pure dermatomyositis
	OM	Overlap myositis: with at least one clinical overlap feature and/or a myositis autoantibody^c^
	CAM	Cancer-associated myositis: with clinical paraneoplasic features and without a myositis autoantibody or anti-Mi-2

^a^Clinical overlap features: arthritis, Raynaud's phenomenon, sclerodactyly, scleroderma proximal to metacarpophalangeal joints, typical systemic sclerosis-type calcinosis in the fingers, lower esophageal or small bowel hypomotility, lung involvement (carbon monoxide diffusing capacity <70% of the normal predicted value, restrictive syndrome, and/or interstitial lung disease on chest radiogram or computed tomography scan), discoid lupus, anti-native DNA antibodies plus hypocomplementemia, four or more of 11 American College of Rheumatology systemic lupus erythematosus criteria, and antiphospholipid syndrome. ^b^Clinical paraneoplasic features: cancer within 3 years of myositis diagnosis, plus absence of multiple clinical overlap features, plus, if cancer was cured, myositis was cured as well. ^c^Anti-synthethases (Jo-1, PL-7, PL-12, OJ, EJ, and KS), systemic sclerosis autoantibodies (centromere protein [CENP]-B, DNA topoisomerase I, RNA polymerase III, and Th/To), autoantibodies commonly associated with systemic sclerosis in overlap (U1RNP, U2RNP, U3RNP, U5RNP, PM-Scl, and Ku), anti-SRP (signal recognition particle), and anti-nucleoporins. Autoantibodies to Mi-2, Ro, and La are not included. Modified from Troyanov and coworkers [14].

### AIM classifications

Patients were categorized at AIM diagnosis according to three classifications, as shown in Table 1: the original Bohan and Peter classification [3,4], a modified Bohan and Peter classification developed by us [14], and a clinicoserologic classification also developed by us [14]. The distribution of patients using the modified Bohan and Peter classification was done before results of AIM autoantibody testing were available. Diagnosis of an associated CTD was made according to the American College of Rheumatology classification criteria for systemic lupus erythematosus (SLE), rheumatoid arthritis, and systemic sclerosis (SSc) [14]. Before AIM diagnosis, 16 patients fulfilled American College of Rheumatology criteria for another CTD (seven SSc, six rheumatoid arthritis, and three SLE patients), whereas at AIM diagnosis eight additional patients fulfilled such criteria (mostly SSc).

### Panel of 21 autoantibodies tested and screening strategy

Serum samples were coded and frozen at -80°C. The timing of serum samples relative to the diagnosis of myositis was as follows: nine sera were obtained at least 6 months before AIM diagnosis, 45 sera were obtained at diagnosis, and 46 sera were obtained at least 6 months after diagnosis, with 23 of those more than 5 years after diagnosis.

The following MSAs and MAAs were studied. Anti-synthetasesencompassed Jo-1, OJ, EJ, KS, PL-7, and PL-12 aAb specificities [15-17]. SSc autoantibodies included autoantibodies to centromere protein (CENP)-B, DNA topoisomerase I (topo; Scl-70), Th/To (Th), and RNA polymerase III (RNAPOLIII) [18-20]. Autoantibodies commonly associated with SSc in overlap encompassed autoantibodies to PM-Scl, U1RNP, U2RNP, fibrillarin, U5RNP, and Ku autoantigens [21-26]. Myositis autoantibodies also included anti-signal recognition particle (SRP) [27] and anti-nucleoporins [28,29]. Anti-Mi-2 (which are DM specific when determined by immunodiffusion or immunoprecipitation [IPP] and are not associated with overlap manifestations) [30], as well as anti-Ro(SS-A) and anti-La (SS-B; which are commonly associated with MSAs and MAAs), were also tested for [14]. The prevalence of autoantibodies was determined by systematic application of the methods that follow to all sera.

#### Indirect immunofluorescence

Antibodies to centromeres and nucleoporins were detected by indirect immunofluorescence on HEp-2 cells (Antibodies Inc., Davis, CA, USA) [18,28].

#### Addressable laser bead immunoassay

Microspheres embedded with laser-reactive dyes (Luminex Corporation, Austin, TX, USA), coupled with native Jo-1, U1RNP, topo, La, and Sm antigens from calf thymus, or a mixture of native Ro from calf thymus and recombinant Ro52 antigens, were part of a commercial kit (QuantaPlex™ SLE Profile 8; INOVA Diagnostics Inc., San Diego, CA, USA). Addressable laser bead immunoassay (ALBIA) allowed semiquantitative detection of autoantibodies to Jo-1, U1RNP, topo, Ro, La, and Sm. The assay was performed at the Advanced Diagnostics Laboratory of the University of Calgary. Briefly, each test serum was diluted to 1:1,000, and 50 μl was added to a well of a microtiter plate, mixed with the antigen-coated beads that were preserved in the well, and incubated for 30 min. Then, 50 μl of phycoerythrin-conjugated goat anti-human immunoglobulin G (γ-chain specific; Jackson ImmunoResearch, Inc., West Grove, PA, USA) was added to each well and incubated for an additional 30 min. The reactivity of the antigen-coated beads was determined on a Luminex 100 dual-laser flow cytometer. The antigens are each bound to distinct fluorochrome-labeled microspheres, and this flow cytometer can discriminate the color of each bead from the others as well as measure the fluorescent intensity of the conjugate on each bead [31]. The cut-off for a positive test result was based on the reactivity of control samples. The control samples included in the kit were titrated to provide high, medium, low, and negative values [32].

#### Line immunoassay

LIA was performed at the Advanced Diagnostics Laboratory by Euroline-WB assay (Euroimmun AG, Luebeck, Germany). Test strips are coated with sodium dodecyl sulphate extracted and electrophoretically separated whole HeLa cell proteins that are transferred to nitrocellulose strips that then allow detection of autoantibodies against Mi-2, Ku-72, and Ku-86 autoantigens using a conventional immunoblot protocol. Each strip also contains nitrocellulose chips on which recombinant antigens (PM-Scl, PL-7, and PL-12) and native Jo-1 purified by affinity chromatography were individually applied. The recombinant PM-Scl was a full length (100 kDa) PM-Scl derived from a human cDNA expressed in baculovirus-infected insect cells. The specificity of the reactivities was validated by using known positive and negative controls. Using this LIA and sera from 70 patients with AIM, the following autoantibody frequencies were observed: 6% anti-Mi-2, 14% anti-PM-Scl, 10% anti-Jo-1, 6% anti-PL-7, 3% anti-PL-12, and 9% anti-Ku. These autoantibodies were not observed in patients with SLE (*n *= 30; except for anti-PL-7 in none patient) or SSc (*n *= 20) or in healthy blood donors (*n *= 50; data available online [33]). In addition, anti-Mi-2 was not detected in sera from 100 normal control individuals and 100 SLE patients at the Advanced Diagnostics Laboratory of the University of Calgary [32] (unpublished data).

#### Enzyme-linked immunosorbent assays

Anti-RNAPOLIII autoantibodies were detected by enzyme linked immunosorbent assay (ELISA) using a recombinant RNAPOLIII fragment containing the immunodominant epitope (MBL Co., Nagoya, Japan) [20]. Positive controls were anti-RNAPOLIII SSc sera provided by M Kuwana (Keio University School of Medicine, Japan) [20]. The cut-off value was 11 units/ml, as recommended by the manufacturer. Anti-topo autoantibodies were detected by ELISA using native full-length topo extracted and purified from calf thymus (Immunovision. Springdale, AR, USA) [34]. For sera positive for anti-Ro by ALBIA, the specificity for anti-Ro52 and anti-Ro60 was further determined by ELISA using recombinant human Ro52 expressed in *Escherichia coli *and native Ro60 from calf thymus (INOVA Diagnostics Inc., San Diego, CA, USA). For sera positive for anti-centromere autoantibodies by indirect immunofluorescence, reactivity with CENP-B was confirmed by ELISA using recombinant full-length CENP-B from baculovirus-infected Sf9 cells (Diarect AG, Freiburg, Germany) [35].

#### Anti-fibrillarin assay

Sera were screened for anti-fibrillarin by ALBIA using purified recombinant fibrillarin protein (Mikrogen GmbH, Neuried, Germany) and test serum diluted to 1:1,000 [36]. Control negative and standard positive sera were included in each assay. The presence of anti-fibrillarin was confirmed by translation and transcription (TNT) of a full-length cDNA and IPP of the radiolabeled recombinant protein [37,38]. This assay was initially validated in experimental autoimmunity and shown to have greater than 90% specificity and 8% sensitivity for SSc [39].

#### Protein A assisted IPP

IPP was performed by one of us (INT) for both nucleic acid and protein analyses, along with double immunodiffusion as described elsewhere [14-17]. Autoantibodies identified using these tests include all of the described anti-synthetases, anti-RNA polymerase III, anti-Th, anti-U2RNP, anti-U3RNP, anti-U5RNP, and anti-SRP [14-17]. For IPP, nucleic acid analysis used 3 to 5 mg of protein A-sepharose, 20 μl of patient serum, and unlabeled HeLa cell extract (>10^6 ^cells). Immunoprecipitates were analyzed by 7 to 8 mol/l urea and 10% polyacrylamide gel electrophoresis with silver stain development. Protein analysis used 1 to 2 mg protein A-sepharose, 10 to 15 ml serum, and 35S-methionine-labeled HeLa cell extract (>10^5 ^HeLa cells). Immunoprecipitates were analyzed by sodium dodecyl sulphate polyacrylamide gel electrophoresis (between 8% and 10%) [14-17].

### Statistical analyses

Associations between categorical variables and comparisons of the frequency of a given autoantibody between mutually exclusive subsets of patients were based on two-tailed χ^2 ^tests. The frequency of OM versus other AIM diagnoses used was analyzed using McNemar's test for comparing groups of paired samples. To assess the relationships between a given autoantibody and clinical manifestations, we employed multiple logistic regression with forward selection of independent variables. A separate multivariable logistic regression model was obtained for each autoantibody, with a binary indicator of its presence/absence as the dependent variable, and with clinical characteristics as the candidate independent variables. Adjustment for age and sex was also considered. To obtain parsimonious multivariable models, and ensure the critical ratio of at least five 'outcomes' (here presence of the respective autoantibody) per independent variable in the model, the most significant variables were entered into the model one at a time as long as the corresponding *P *value (from the two-tailed Wald χ^2 ^test) did not exceed 0.05. Final results are reported as adjusted odds ratios with 95% confidence intervals for those variables that were included in the final model. In all analyses, two-tailed *P *< 0.05 was considered statistically significant. Analyses were performed using SAS 9.1 statistical software package (SAS Institute Inc, Cary, NC, USA).

## Results

### Frequency of autoantibodies to 21 autoantigens

**Figure 1:**
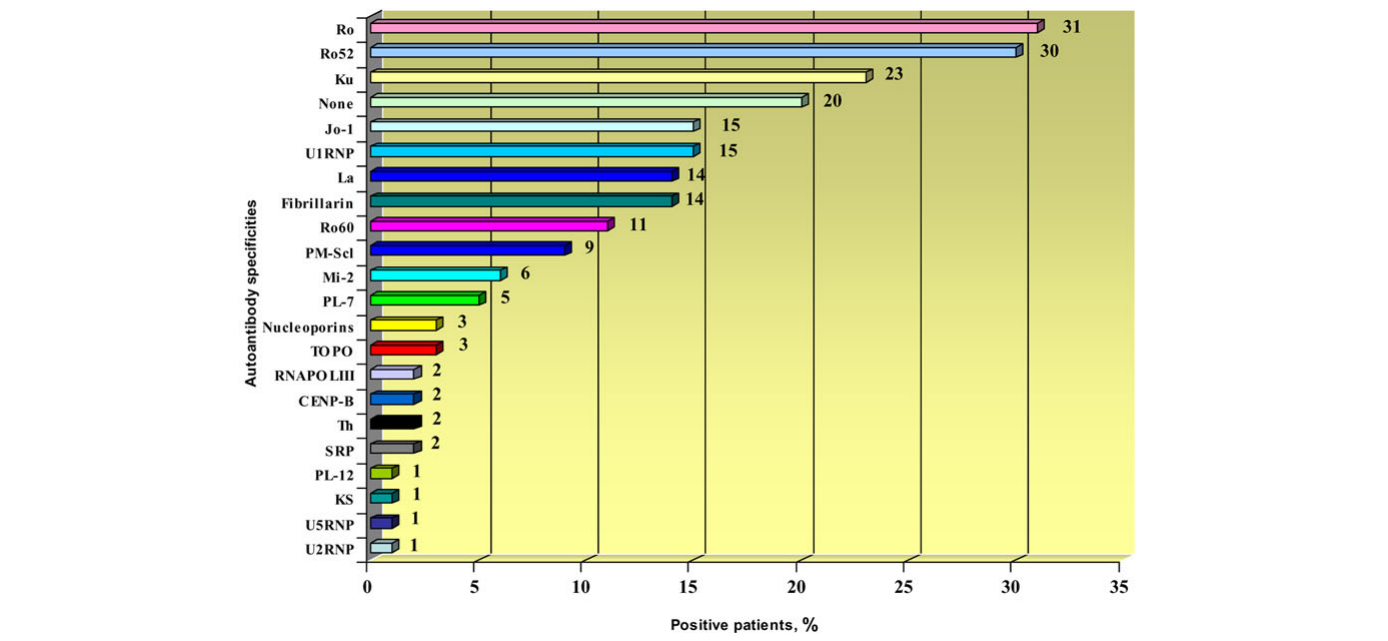
Frequency of serum autoantibodies to 21 autoantigens in 100 French Canadian patients with autoimmune myositis. Autoantibodies were observed to 19 (90%) of the specificities tested. Anti-OJ and anti-EJ (both anti-synthetases) were not detected. One or more autoantibodies were present in 80% of patients. Autoantibodies to synthetases (Jo-1, PL-7, PL-12, and KS) and systemic sclerosis autoantibodies were present overall in 22% and 9% of patients, respectively. The overall frequency is over 100% because 44% of patients had more than one autoantibody. Anti-Ro were determined by ALBIA whereas anti-Ro52 and anti-Ro60 fine specificities were identified by ELISA. See Materials and methods (in the text) for a description of immunoasssays. ALBIA, addressable laser bead immunoassay; CENP, centromere protein; ELISA, enzyme-linked immunosorbent assay; RNAPOLIII, RNA polymerase III; SRP, signal recognition particle; TOPO, topoisomerase I.

The frequency of each autoantibody in the cohort is shown in Figure 1. Two features are noteworthy. First, the overall frequency of autoantibodies, as detected using various methods, was high, with 80% (80 patients) expressing one or more autoantibody. In contrast, in our previous report using the same serum samples [14] only 56% of the patients expressed one or more autoantibodies. Second, the diversity of autoantibodies was also high, as indicated by the detection of autoantibodies to 19 of the 21 (90%) autoantigens tested. The most common autoantibody, present in 31% (31 patients), was anti-Ro (detected using ALBIA). Further analysis by ELISA for fine specificity revealed that anti-Ro52 autoantibodies was most common, occurring in 97% (30/31 patients) of anti-Ro positive sera, whereas anti-Ro60 autoantibodies were present in 35.5% (11/31 patients; Figure 1). Almost all sera with anti-Ro60 (91%; 10/11 patients) displayed anti-Ro52 as well, confirming that among patients with AIM the most common anti-Ro specificity is anti-Ro52 [40]. The next most common autoantibodies were anti-Ku (23%; detected using LIA), anti-Jo-1 (15%), anti-U1RNP (15%), and anti-fibrillarin and anti-La (each 14%; Figure 1).

Of the 15 remaining potential autoantibody specificities, 13 specificities were present with frequencies ranging from 9% (anti-PM-Scl) to 1% (for instance, anti-KS, anti-U2RNP, and anti-U5RNP; Figure 1). Anti-Mi-2 and anti-nucleoporins were detected in 6% and 3% of patients, respectively. Only anti-OJ and anti-EJ (both anti-synthetases) were not detected.

### Frequency of anti-synthetases and SSc autoantibodies

Anti-synthetases were present overall in 22% (22 patients; Figure 1). Anti-Jo-1 and anti-PL-7 were the most common specificities, accounting for 68% (15/22 patients) and 23% (5/22 patients) of anti-synthetases, respectively, whereas anti-PL-12 and anti-KS were rare (one patient each). SSc autoantibodies (anti-CENP, anti-topo, anti-Th, and anti-RNAPOLIII) were uncommon (9%).

### Multiple myositis autoantibodies frequently coexist

Table 2 shows that among the 80 patients with AIM autoantibodies, multiple autoantibodies were found in 44 (55%) patients. Thus, in the presence of the more common autoantibodies (such as to Ku, synthetases, U1RNP, fibrillarin, PM-Scl, or SSc autoantigens), at least one more autoantibody was present in the majority (mean 78.5%, range 60% to 93%) of sera (Table 2). For example, of 22 patients with autoantibodies to synthetases, 18 (82%) expressed one or more additional autoantibodies. Furthermore, in the presence of the more common autoantibodies, at least two more autoantibodies were present in 20% to 65% (mean 43.3%) of sera. For example, 10 out of 22 (46%) of patients with autoantibodies to synthetases expressed two or more additional autoantibodies. The largest number of concurrent autoantibodies was observed in a single serum with six autoantibodies. Four additional patient sera displayed four specificities. Overall, not less than 44 distinct combinations of autoantibodies were counted. These data highlight that a major subset of AIM is characterized by the simultaneous presence of two or more autoantibodies rather than by single specificities.

**Table 2:** Frequency of multiple autoantibodies in 100 patients with autoimmune myositis

Additional antibodies^a^	Autoantibodies											Patients (total)	
	
	Ku (*n *= 23)	tRNA (*n *= 22)	U1RNP (*n *= 15)	Fibrillarin (*n *= 14)	PM-Scl (*n *= 9)	SSc (*n *= 9)	NUP (*n *= 3)	SRP (*n *= 2)	Ro (*n *= 31)	La (*n *= 14)	Mi-2 (*n *= 6)	No antibody (*n *= 20)	= 1 antibody (*n *= 80)
None	7 (31)	4 (18)	6 (40)	1 (7)	2 (22)	1 (11)	2 (67)	2 (100)	4 (13)	3 (21)	3 (50)	0	36 (36)^b^
1 more	7 (31)	8 (36)	6 (40)	4 (28)	4 (44)	3 (33)	1 (33)	0	12 (39)	4 (29)	2 (33)	0	26 (26)
2 more	5 (21)	6 (28)	2 (13)	6 (43)	1 (12)	3 (33)	0	0	10 (32)	4 (29)	0	0	12 (12)
≥3 more	4 (17)	4 (18)	1 (7)	3 (22)	2 (22)	2 (23)	0	0	5 (16)	3 (21)	1 (17)	0	6 (6)

Values are expressed as number (%). tRNA synthetases (tRNA) include Jo-1, PL-7, PL-12, and KS. Systemic sclerosis (SSc) autoantigens include topoisomerase I, RNA polymerase III, centromere protein B, and Th. ^a^Categories are mutually exclusive. ^b^Includes an additional patient who had anti-U5RNP (not shown). NUP, nucleoporins; RNP, ribonucleoprotein; SRP = signal recognition particle.

### Associations and exclusions between myositis autoantibodies

As shown in Table 3, mutual exclusion was noted between autoantibodies to synthetases, Mi-2, and SRP, as reported previously [3].

**Table 3:** Patterns of associations between single autoantibodies in 100 patients with autoimmune myositis

	Ro	Ku	Jo-1	PL-7	PL-12	KS	U1RNP	Fibrillarin	La	PM-Scl	Topo	RNAPOLIII	CENP-B	Th	Mi-2	NUP	SRP	U5RNP	U2RNP
*n*	31	23	15	5	1	1	15	14	14	9	3	2	2	2	6	3	2	1	1
Methods of detection	ALBIA	LIA, IPP	LIA, IPP, ALBIA	LIA, IPP	LIA, IPP	IPP	IPP, ALBIA	TNT, ALBIA	ALBIA, ELISA	LIA, IPP	ALBIA, ELISA	IPP, ELISA	ELISA, IIF	IPP	LIA, IPP	IPP	IPP	IPP	IPP
Ro	**4**	6	11	1	1	1	6	6	5	6	2	-	1	1	-	-	-	-	-
Ku	6	**7**	4	1	-	-	2	8	4	2	-	1	-	-	1	-	-	-	-
Jo-1	11	4	**2**	-	-	-	-	2	3	1	2	-	1	-	-	-	-	-	-
PL-7	1	1	-	**2**	-	-	-	1	2	-	-	-	-	-	-	-	-	-	-
PL-12	1	-	-	-	**-**	-	-	1	-	-	-	-	-	-	-	-	-	-	-
KS	1	-	-	-	-	**-**	-	-	-	-	-	-	-	-	-	-	-	-	-
U1RNP	6	2	-	-	-	-	**6**	2	2	-	-	-	-	1	-	-	-	-	1
Fibrillarin	6	8	2	1	1	-	2	**1**	2	1	-	1	1	-	1	-	-	-	-
La	5	4	3	2	-	-	2	2	**3**	1	1	-	-	-	1	1	-	-	1
PM-Scl	6	2	1	-	-	-	-	1	1	**2**	1	-	-	-	1	-	-	-	-
Topo	2	-	2	-	-	-	-	-	1	1	**-**	-	-	-	1	-	-	-	-
RNAPOLIII	-	1	-	-	-	-	-	1	-	-	-	**-**	-	-	1	-	-	-	-
CENP-B	1	-	1	-	-	-	-	1	-	-	-	-	**-**	-	-	-	-	-	-
Th	1	-	-	-	-	-	1	-	-	-	-	-	-	**1**	-	-	-	-	-
Mi-2	-	1	-	-	-	-	-	1	1	1	1	1	-	-	**3**	-	-	-	-
NUP	-	-	-	-	-	-	-	-	1	-	-	-	-	-	-	**2**	-	-	-
SRP	-	-	-	-	-	-	-	-	-	-	-	-	-	-	-	-	**2**	-	-
U5RNP	-	-	-	-	-	-	-	-	-	-	-	-	-	-	-	-	-	**1**	-
U2RNP	-	-	-	-	-	-	1	-	1	-	-	-	-	-	-	-	-	-	**-**

Numbers in bold denote that a single autoantibody specificity was detected. -, absence of autoantibody; ALBIA, addressable laser bead immunoassay; CENP, centromere protein; ELISA, enzyme-linked immunosorbent assay; IIF, indirect immunofluorescence; IPP, immunoprecipitation; LIA, line immunoassay; NUP, nucleoporins; RNAPOLIII, RNA polymerase III; SRP, signal recognition particle; TNT, transcription and translation assay; Topo, topoisomerase I.

#### Anti-Ro, anti-Ro52, and anti-Ro60

Anti-Ro autoantibodies were commonly associated with other specificities (67%; 12/18 specificities), most commonly autoantibodies to synthetases (45%; 14/31), notably Jo-1 (35%; 11/31), U1RNP (19%; 6/31), and Ku, fibrillarin and PM-Scl (each 19%; 6/31). All anti-Ro positive patients with anti-Ku, anti-fibrillarin, and anti-PM-Scl had anti-Ro52.

#### Anti-Ku

The most frequently associated autoantibodies were anti-fibrillarin (35%), anti-Ro (26%), and anti-Jo-1 (17%).

#### Anti-fibrillarin

These were associated with 11 of the 18 (61%) other specificities, most commonly anti-Ku (57%; 8/14).

#### Anti-synthetases

The frequency of anti-Ro was greater among patients with anti-synthetases (64%; 14/22) than among all other patients (22%; 17/78; odds ratio 6.8, 95% confidence interval 2.2 to 17.4; *P *= 0.0004). All patients with anti-synthetases and anti-Ro exhibited anti-Ro52 autoantibodies as well (14/14), as compared with 35% (5/14) for anti-Ro60. Anti-synthetases were also associated with anti-Ku (32%; 7/22) and anti-fibrillarin (18%; 4/22). Anti-Jo-1 autoantibodies were associated with autoantibodies to Ro (73%; 11/15), Ku (27%), La (20%), fibrillarin (13%), topo (13%), PM-Scl (7%), and CENP-B (7%; Table 3).

#### Anti-Mi-2

Anti-Mi-2 autoantibodies detected by IPP only (anti-Mi-2-IPP; 3) were not associated with other autoantibodies, whereas anti-Mi-2 autoantibodies detected by LIA only (anti-Mi-2-LIA; 3) were associated with other autoantibodies (Table 3).

### Fine specificity of anti-Ku autoantibodies

All three anti-Ku positive sera by IPP reacted only with the Ku-86 peptide in the LIA. Of the 20 anti-Ku negative sera by IPP, nine (45%) reacted only with the Ku-86 peptide by LIA, five (25%) reacted with the Ku-72 peptide only, and six (30%) reacted with both peptides. This suggests that autoantigen presentation in the LIA procedure revealed additional linear or cryptic epitopes [41] or that stringent conditions used during IPP may alter antigen binding.

### Comparison of immunoassay sensitivities

For several autoantibodies, sensitivities concurred; for instance, the 15 sera positive for anti-Jo-1 by IPP were positive by LIA in all instances and by ALBIA in 14 (93.3%) cases. Similarly, sera with anti-U1RNP detected by IPP were also positive by ALBIA, and sera with anti-fibrillarin by ALBIA were also positive by TNT assay. Anti-PL-12 autoantibodies were detected by both IPP and LIA. However, a discrepancy in sensitivity was noted for anti-Ku, which was detected in 23 patients by LIA but in only three (13%) of these patients by IPP. Finally, of the six sera with anti-Mi-2, three were anti-Mi-2-IPP only and three anti-Mi-2-LIA only.

### Demographics, distribution of patients, and associated autoantibodies according to the modified Bohan and Peter classification at diagnosis

Most patients were female (female to male ratios: 13:1 for PM, 18:5 for DM, 41:19 for OM, and 3:0 for cancer-associated myositis [CAM]). As shown in Table 4, autoantibodies to Ro, Ku, fibrillarin, synthetases, U1RNP, La, PM-Scl, and nucleoporins were not restricted to a single diagnostic category. However, anti-Mi-2-IPP were restricted to patients with DM rashes (two patients with DM, and one patients with CAM associated with a DM rash), whereas anti-Mi-2-LIA (three patients) were associated with OM. None of the patients with anti-Mi-2-LIA developed DM rashes at follow up (mean duration 9.98 years, range 9.2 to 10.4 years). Overall, OM accounted for the majority (65% to 86%) of these various specificities, but only anti-fibrillarin and anti-U1RNP were more common in OM than in all other myositis patients (*P *= 0.007 and *P *= 0.024, respectively; Table 4). In contrast, the frequency of anti-synthetases (22 patients) was similar in PM (21.4%), DM (17.4%), and OM (25%) patients (*P *= 0.46 for OM versus all others). Anti-PM-Scl were present in both DM (three patients) and OM (six patients; *P *= 0.73). Overall, except for anti-SRP and anti-Mi-2, none of these autoantibodies segregated with a unique AIM diagnostic category. Finally, the mean number (and range) of autoantibodies in each diagnostic category was as follows: 1.81 (0 to 6) for OM, 1.13 (0 to 3) for DM, 0.92 (0 to 3) for PM, and 0.33 (0 to 1) for CAM (*P *= 0.0055 by Kruskall-Wallis test for difference of means, excluding CAM). Thus, the greatest mean number of autoantibodies was observed in OM. Interestingly, absence of autoantibodies was associated with significantly decreased risk for OM (Table 4).

**Table 4:** Demographics, associated autoantibodies, and distribution of 100 patients with autoimmune myositis according to the modified Peter and Bohan classification at diagnosis

	PM (*n *= 14)	DM (*n *= 23)	OM (*n *= 60)	CAM (*n *= 3)	*P*^a^
Age at diagnosis (years; mean ± SD)	52.7 ± 16.8	45.4 ± 16.7	45.6 ± 13.5	56.3 ± 11	0.303
Mean follow-up period (years; mean ± SD)	8.15 ± 4.8	12.52 ± 9.2	8.05 ± 6.1	7.64 ± 2.7	0.013
Associated autoantibodies					
Anti-Ro (*n *= 31)	4 (28.6)	5 (21.7)	22 (36.7)	0	0.185
Anti-Ku (*n *= 23)	3 (21.4)	5 (21.7)	15 (25)	0	0.633
Anti-synthetases (*n *= 22)	3 (21.4)	4 (17.4)	15 (25)	0	0.463
Anti-U1RNP (*n *= 15)	0	2 (8.7)	13(21.7)	0	0.024^b^
Anti-fibrillarin (*n *= 14)	0	1 (4.3)	13 (21.7)	0	0.007^c^
Anti-La (*n *= 14)	1 (7.1)	3 (13)	10 (16.7)	0	0.394
SSc autoantibodies (*n *= 9)	0	1 (4.3)	8 (13.3)	0	0.081
Anti-PM-Scl (*n *= 9)	0	3 (13)	6 (10)	0	0.737
Anti-Mi-2-IPP (*n *= 3)	0	2 (8.7)	0	1 (33)^d^	-
Anti-Mi-2-LIA (*n *= 3)	0	0	3 (5)	0	-
Anti-NUP (*n *= 3)	1 (7.1)	0	2 (3.3)	0	1
Anti-SRP (*n *= 2)	0	0	2 (3.3)	0	0.515
None (*n *= 20)	5(35.7)	6 (26.1)	7(11.7)	2 (66)	0.02^e^

Unless otherwise specified, values are expressed as number (%). Anti-Ro was detected using addressable laser bead immunoassay; for anti-Mi-2-IPP and anti-Mi-2-LIA, autoantibody was present only by immunoprecipitation or line immunoassay. ^a^For Associated autoantibodies: to avoid more than 20% of values being <5, comparisons were made by Fisher's exact test between OM and all others. ^b^Odds ratio 5.2, 95% confidence interval 1.1 to 24.7. ^c^Odds ratio 10.8, 95% confidence interval 1.35 to 86. ^d^The patient also had a DM rash. ^e^Odds ratio 0.27, 95% confidence interval 0.1 to 0.77. CAM, cancer-associated myositis; DM, pure dermatomyositis; OM, overlap myositis; PM, pure polymyositis; SD, standard deviation.

### Impact of autoantibodies on myositis classification at diagnosis

Because of the increased frequency of autoantibodies in the present study in comparison with our previous study [14], we used the novel AIM clinicoserologic classification to compare the distribution of patients according to diagnosis in the current versus the previous reports. As shown in Table 5, the frequency of OM rose from 68% to 82%, not including anti-Ro and anti-Mi-2, whereas the frequency of other diseases decreased to 18% (versus the previous study: by McNemar test, *P *< 0.001; versus the modified Bohan and Peter classification, *P *< 0.001). This increase in the frequency of OM was due to newly detected autoantibodies to Ku (eight), Ro (five), PM-Scl (four), synthetases (three), U1RNP (two), and fibrillarin (one). Of note is the overall decrease in PM frequency from 45% using the original Bohan and Peter classification to only 7% using the clinicoserologic classification (Table 5). Taken together, these data support previous observations that OM is the most common AIM, and PM is the least common AIM [14].

**Table 5:** Distribution of 100 patients at myositis diagnosis according to novel classifications for autoimmune myositis

Classifications	PM	DM	OM	CAM	Total
Original Peter and Bohan classification: previous study^a^	45	28	24	3	100
Modified Peter and Bohan classification: previous study	14	23	60	3	100
Novel clinicoserologic classification: previous study^b^	10	20	68	2	100
Novel clinicoserologic classification: present study^b^	7	8	82	3	100

Values are expressed as percentages. Frequency of OM versus non-OM by McNemar's test for comparing groups of paired samples: modified versus original Bohan and Peter classifications, χ^2 ^= 34.03, *P *< 0.001; novel clinicoserologic classification (previous study) versus modified classification, χ^2 ^= 6.12, *P *= 0.013; novel clinicoserologic classification (present study) versus modified classification: χ^2 ^= 20.04, *P *< 0.001; and novel clinicoserologic classifications (present study versus previous study), χ^2 ^= 12.07, *P *< 0.001. ^a^Data from Troyanov and coworkers [14]. ^b^Excluding anti-Ro and anti-Mi-2. CAM, cancer associated myositis; DM, pure dermatomyositis; OM, overlap myositis; PM, pure polymyositis.

### Clinical features independently associated with AIM autoantibodies

As shown in Table 6, interstitial lung disease, arthritis, fever, and puffy hands (but neither mechanic's hands nor Raynaud's) were associated with a higher frequency of anti-Jo-1. In contrast, both Raynaud's and lung involvement were associated with anti-fibrillarin (Table 6). Raynaud's, arthritis, and sclerodactyly were strongly associated with anti-U1RNP. Telangiectasias, sclerodactyly, and sclerodermatous skin proximal to the MCP joints were associated with SSc autoantibodies. A decreased risk for an associated American College of Rheumatology criteria defined CTD was linked to anti-Jo-1, whereas an increased risk was linked with anti-fibrillarin and anti-U1RNP (Table 6).

**Table 6:** Independent associations of autoantibodies with specific sets of clinical features, therapeutic outcomes, and myositis course by stepwise multiple logistic regression in 100 patients with autoimmune myositis

Autoantibodies	Clinical features^a^	OR	95% CI	*P*
Anti-Jo-1^b^	Interstitial lung disease	14.5	3.52 to 59.78	<0.001
	Arthritis	11.6	2.92 to 45.65	<0.001
	Fever	9.7	2.72 to 34.91	<0.001
	Puffy hands	9.6	2.82 to 32.92	<0.001
	Associated CTD	0.14	0.03 to 0.70	0.016
	Response to prednisone alone	0.04	0.01 to 0.32	0.002
	Need for second line drug	25.3	4.24 to 150	<0.001
Anti-fibrillarin	Raynaud's phenomenon	10.9	2.83 to 42.60	<0.001
	Any lung involvement	3.5	1.01 to 12.39	0.050
	Associated CTD	4.1	1.14 to 14.50	0.031
Anti-U1RNP	Raynaud's phenomenon	9.8	2.28 to 42.59	0.002
	Arthritis	5.8	1.74 to 19.18	0.004
	Sclerodactily	6.8	2.03 to 22.49	0.001
	Associated CTD	6.8	1.76 to 26.55	0.006
	Associated SSc	6.1	1.83 to 20.26	0.003
	Monophasic myositis course	8.1	1.39 to 46.7	0.020
SSc autoantibodies	Telangiectasias	19.3	2.05 to 181.51	0.010
	Sclerodactyly	8.6	1.88 to 39.60	0.005
	Scleroderma proximal to MCP	5.3	1.07 to 26.78	0.041
	Associated SSc	5.8	1.29 to 25.78	0.022
Anti-Ro or anti-Ro52	Response to prednisone alone	6.2	1.23 to 31.64	0.027
	Need for second line drug	0.12	0.03 to 0.54	0.006

Clinical associations were determined at myositis diagnosis, whereas associations with specific myositis courses and therapeutic responses were determined at last follow up. Results were very similar when adjusted for age and sex. See Materials and methods (in the text) for definitions of clinical findings. ^a^Definitions were previously described by Troyanov and coworkers [14]. ^b^Anti-Jo-1 (or anti-synthetases overall) were also associated with higher serum creatine kinase at myositis diagnosis (OR 5.3; *P *< 0.0001). CI, confidence interval; CTD, connective tissue disease; DM, dermatomyositis; MCP, metacarpophalangeal joints; OR, odds ratio; SSc, systemic sclerosis.

### Association between therapeutic outcomes, myositis course, and autoantibodies

Table 6 also shows that specific patterns of myositis responsiveness to standard therapy are independently associated with certain autoantibodies. Thus, a decreased response to prednisone alone and an increased need for a second-line immunosuppressive drug were highly associated with anti-Jo-1. In contrast, myositis responsive to prednisone alone and a reduced need for a second-line drug were associated with both anti-Ro and anti-Ro52 (Table 6). Finally, a monophasic course of myositis was associated with anti-U1RNP.

## Discussion

Several conclusions stem from the present study.

### When using newly developed assays such as ALBIA and LIA, the prevalence of autoantibodies in patients with AIM is much higher than previously appreciated

Our report is the first to reassess the prevalence of autoantibodies in the same serum samples obtained from the same AIM cohort. Thus, 80% of patients expressed one or more autoantibodies in the present report, whereas in the previous report the frequency was only 56% [14]. The most common autoantibody overall was anti-Ro52, which was present in 30% of patients, followed by anti-Ku (23%), and anti-Jo-1 and anti-U1RNP (both 15%). Inter-cohort differences in the frequency of autoantibodies have been reported. For example, whereas the frequency of autoantibodies was only 53% in a study of European myositis patients, it was 74% and 80% in Italian and Polish cohorts, respectively [42-44]. However, to our knowledge, a systematic study of intra-cohort differences has not been reported.

### The higher frequency of autoantibodies is due to an increased sensitivity of the detection methods employed

Certain autoantibodies were detected for the first time in 24 patients. The new specificities detected were Ku (12 patients), fibrillarin (five), Ro (five), synthetases (three; all PL-7), PM-Scl (three), and U1RNP (three). In our previous report, using a highly specific IPP method, the frequency of anti-Ku was only 2% [14]. However, in the present study, we used a more sensitive LIA method. Indeed, immunoblotting has been associated with a 16% to 33% frequency of anti-Ku in OM patients [17,43,45]. Similarly, a 19% frequency of anti-Ku by immunoblotting was reported in PM and DM [41]. Ethnicity/genetic background also influences the anti-Ku immune response, because the highest frequency of anti-Ku has been reported in Japanese and African American patients [17,46,47]. A combination of historical and geographic factors has resulted in relative genetic homogeneity in French Canadian patients, with ensuing potential modulation of autoimmune responses [18,48].

Anti-fibrillarin was not detected by IPP in our previous report, although the IPP method used was not optimized for the detection of this antibody [14]. In the present report we screened for anti-fibrillarin by ALBIA using purified recombinant fibrillarin protein, yielding an autoantibody frequency of 14%. All positive anti-fibrillarin results were confirmed by highly specific, protein-based TNT assay [37,38]. Anti-fibrillarin autoantibodies are uncommon in conditions other than SSc [23,24,46]. Interestingly, by multiple logistic regression we found that the presence of an associated CTD, most commonly SSc, was linked to anti-fibrillarin. Furthermore, the association between anti-fibrillarin and myositis as well as lung involvement has previously been reported in SSc patients [24,49]. Finally, as mentioned for anti-Ku, immunogenetics may have influenced anti-fibrillarin autoimmune response in our French Canadian patients [18,48].

### A major subset of AIM is characterized by complex associations of autoantibodies and extremely marked serologic heterogeneity

Our data expand on previous studies of autoantibodies in AIM sera and show that the diversity of autoantibodies in AIM sera is high and their mutual associations are complex [50]. In particular, anti-Jo-1 was almost always associated with autoantibodies to one or more of seven autoantigens, notably anti-Ro52. No less than 44 distinct combinations of autoantibodies were identified, indicating remarkable heterogeneity of B-cell responses in AIM. Such polyreactivity is common in other CTDs such as SLE. However, polyreactivity has been observed in only 0.8% of sera submitted to a clinical laboratory for routine autoantibody analysis by ALBIA [51], suggesting that polyreactivity is related to the primary CTD diagnosis. Thus, clinicians should be aware that several autoantibodies may be present in single AIM patient sera and that identification of a common autoantibody specificity (for instance, anti-Ro) does not preclude the presence of other autoantibodies that may have useful diagnostic, prognostic, and even therapeutic significance. This issue is hindered by the fact that several autoantibodies are not routinely detected in most clinical diagnostic laboratories and, as currently constituted, are relatively costly and labor intensive. Moreover, as ALBIA used herein and other multiplexed autoantibody assays become more widely available, it will be important for clinicians to become aware that myositis subsets often are not single autoantibody entities [32].

Taken together, these data suggest that a major subset of AIM is characterized by complex associations of autoantibodies rather than by single specificities. This has potential importance for future studies of the clinical associations and prognostic value of myositis autoantibodies. It will be of interest to dissect further whether there are differences within autoantibody defined groups based on whether they do or do not have a particular set of associated autoantibodies. Furthermore, these data provide a basis and a rationale for patient profiling and its application to therapeutics, because it cannot be assumed that the B-cell response is the same in all or even the majority of patients in a given diagnostic category, as discussed below.

### Myositis autoantibodies are not restricted to a unique myositis diagnostic category

Except for the uncommon anti-SRP and anti-Mi-2 autoantibodies, all autoantibodies occurred in at least two diagnostic categories, mainly OM, DM, and/or PM [8,42,43]. An intriguing issue is the different diagnoses associated with anti-Mi-2 depending on the detection method. Anti-Mi-2 is strongly associated with DM when it is detected by IPP of radiolabeled cell extracts, and our data are in agreement because our patients with anti-Mi-2-IPP had DM rashes [30]. However, such diagnostic specificity was altered when ELISA with different fragments of recombinant Mi-2 was used for screening a large AIM cohort [42]. Similarly, anti-Mi-2-LIA in our cohort was observed only in OM patients. Anti-Mi-2 was recently reported in PM patients with arthritis, Raynaud's phenomenon, and interstitial lung disease, a cluster of manifestations similar to OM according to the modified Bohan and Peter classification [52]. Thus, the clinical significance of anti-Mi-2 appears to depend on the detection method.

In a previous report [53], one of us (INT) focused on the characterization of Mi-2 epitopes. It was shown that only 50% of sera reactive with the 240 kDa major Mi-2 autoantigen immunoprecipitated from HeLa cells also reacted with Mi-2 from HeLa cells on immunoblots. Failure to detect by immunoblotting reactivity with this major 240 kDa Mi-2 protein could have been due to exclusive reaction of these anti-Mi-2 sera with conformational epitopes and not with the denatured proteins used in immunoblots. Moreover, the uniform reactivity with the 240 kDa Mi-2 protein does not exclude additional reactivity of anti-Mi-2 sera with other Mi-2 epitopes. In the present study, given that the LIA uses blotted proteins, it is possible that the discrepant results between LIA and IPP are due to a similar mechanism.

### OM is the most common AIM and PM is least common

Using our proposed clinicoserologic classification to reassess the diagnostic impact of autoantibody results obtained with more sensitive immunoassays, an increase in the frequency of OM in our cohort from 68% to 82% was noted, whereas PM was least common. These drastic changes strengthen OM as the most common AIM [8-11,14].

### Certain myositis autoantibodies are markers for distinct overlap syndromes and predict therapeutic outcomes

We identified statistically significant and independent associations between clinical manifestations and certain myositis autoantibodies. Thus, interstitial lung disease, arthritis, fever, puffy hands, higher serum creatine kinase, a decreased risk for an associated CTD, a reduced myositis response to prednisone alone, and an increased need for a second-line immunosuppressive drug were strongly associated with anti-Jo-1. In contrast, an increased risk for Raynaud's, lung involvement, and an associated CTD were associated with anti-fibrillarin. Raynaud's, arthritis, sclerodactyly, an associated CTD (particularly SSc), and a monophasic myositis course were strongly linked with anti-U1RNP. Finally, myositis responsiveness to prednisone alone and a decreased need for a second-line drug were independently associated with anti-Ro and anti-Ro52.

Taken altogether, these data are consistent with the view that much of AIM is associated with overlap syndrome features [5,11,13,14,43]. Moreover, certain myositis autoantibodies such as anti-Jo-1, anti-fibrillarin, and anti-U1RNP are markers for distinct overlap syndromes, irrespective of their current classification as MSAs or MAAs. Given the independence of the associations identified, the ultimate clinical features and response to therapy in a given myositis patient may be linked to the particular set of associated autoantibodies, and not merely to the presence of a single autoantibody specificity.

### Study limitations

Our study has a number of statistical limitations. It was probably underpowered to unveil clinical associations for the less common autoantibodies or to identify a larger spectrum of associations for the more prevalent autoantibodies (type II error). Conversely, some significant associations observed by multiple regression may be due to type I error. Finally, it is possible that some of the observed associations are the result of ethnicity/geographic and immunogenetic features of our French Canadian cohort. Therefore, our results will need to be confirmed by independent and larger studies of AIM patients from multiple ethnic and geographic areas.

## Conclusion

The prevalence of autoantibodies in patients with AIM is much higher than was previously appreciated. The higher frequency of autoantibodies is due to increased sensitivity of the detection methods employed. A major subset of AIM is characterized by complex associations of autoantibodies and extremely marked serologic heterogeneity. Myositis autoantibodies are not restricted to a unique myositis diagnostic category. Certain myositis autoantibodies are markers for distinct overlap syndromes and predict therapeutic outcomes. The ultimate clinical features, disease course, and response to therapy in a given AIM patient may be linked to the particular set of associated autoantibodies.

## Abbreviations

AIM = autoimmune myositis; ALBIA = addressable laser bead immunoassay; CAM = cancer associated myositis; CENP = centromere protein; CTD = connective tissue disease; DM = dermatomyositis; ELISA = enzyme-linked immunosorbent assay; IPP = immunoprecipitation; LIA = line immunoassay; MAA = myositis associated autoantibody; MSA = myositis specific autoantibody; OM = overlap myositis; PM = polymyositis; RNAPOLIII = RNA polymerase III; SLE = systemic lupus erythematosus; SRP = signal recognition particle; SSc = systemic sclerosis; TNT = translation and transcription; topo = topoisomerase I.

## Competing interests

The authors declare that they have no competing interests.

## Authors' contributions

JLS and MK participated in the design of the study. YT, JLS and MK collected patient data. MK, JLS, MJF and IT performed the experiments and data analysis. MK and JLS wrote the manuscript. JLS supervised the project. All authors read, corrected, and approved the final manuscript.
